# Topological Data Analysis as a Morphometric Method: Using Persistent Homology to Demarcate a Leaf Morphospace

**DOI:** 10.3389/fpls.2018.00553

**Published:** 2018-04-25

**Authors:** Mao Li, Hong An, Ruthie Angelovici, Clement Bagaza, Albert Batushansky, Lynn Clark, Viktoriya Coneva, Michael J. Donoghue, Erika Edwards, Diego Fajardo, Hui Fang, Margaret H. Frank, Timothy Gallaher, Sarah Gebken, Theresa Hill, Shelley Jansky, Baljinder Kaur, Phillip C. Klahs, Laura L. Klein, Vasu Kuraparthy, Jason Londo, Zoë Migicovsky, Allison Miller, Rebekah Mohn, Sean Myles, Wagner C. Otoni, J. C. Pires, Edmond Rieffer, Sam Schmerler, Elizabeth Spriggs, Christopher N. Topp, Allen Van Deynze, Kuang Zhang, Linglong Zhu, Braden M. Zink, Daniel H. Chitwood

**Affiliations:** ^1^Donald Danforth Plant Science Center, St. Louis, MO, United States; ^2^Division of Biological Sciences, University of Missouri, Columbia, MO, United States; ^3^Department of Ecology, Evolution, and Organismal Biology, Iowa State University, Ames, IA, United States; ^4^Department of Ecology and Evolutionary Biology, Yale University, New Haven, CT, United States; ^5^Department of Ecology and Evolutionary Biology, Brown University, Providence, RI, United States; ^6^National Center for Genome Resources (NCGR), Santa Fe, NM, United States; ^7^Department of Crop and Soil Sciences, North Carolina State University, Raleigh, NC, United States; ^8^Department of Plant Sciences, University of California, Davis, Davis, CA, United States; ^9^Vegetable Crops Research Unit, United States Department of Agriculture-Agricultural Research Service (USDA-ARS), Madison, WI, United States; ^10^Department of Horticulture, University of Wisconsin-Madison, Madison, WI, United States; ^11^Department of Biology, Saint Louis University, St. Louis, MO, United States; ^12^Grape Genetics Unit, United States Department of Agriculture-Agricultural Research Service (USDA-ARS), Geneva, NY, United States; ^13^Department of Plant, Food, and Environmental Sciences, Dalhousie University, Truro, NS, Canada; ^14^Department of Plant and Microbial Biology, University of Minnesota – Twin Cities, St. Paul, MN, United States; ^15^Departamento de Biologia Vegetal, Universidade Federal de Viçosa, Viçosa, Brazil; ^16^American Museum of Natural History, New York, NY, United States; ^17^Independent Researcher, Santa Rosa, CA, United States; ^18^Department of Horticulture, Michigan State University, East Lansing, MI, United States; ^19^Computational Mathematics, Science and Engineering, Michigan State University, East Lansing, MI, United States

**Keywords:** leaf shape, leaves, morphology, shape, topology, topological data analysis, persistent homology, morphometrics

## Abstract

Current morphometric methods that comprehensively measure shape cannot compare the disparate leaf shapes found in seed plants and are sensitive to processing artifacts. We explore the use of persistent homology, a topological method applied as a filtration across simplicial complexes (or more simply, a method to measure topological features of spaces across different spatial resolutions), to overcome these limitations. The described method isolates subsets of shape features and measures the spatial relationship of neighboring pixel densities in a shape. We apply the method to the analysis of 182,707 leaves, both published and unpublished, representing 141 plant families collected from 75 sites throughout the world. By measuring leaves from throughout the seed plants using persistent homology, a defined morphospace comparing all leaves is demarcated. Clear differences in shape between major phylogenetic groups are detected and estimates of leaf shape diversity within plant families are made. The approach predicts plant family above chance. The application of a persistent homology method, using topological features, to measure leaf shape allows for a unified morphometric framework to measure plant form, including shapes, textures, patterns, and branching architectures.

## Introduction

Leaves are three-dimensional structures that grow through time, but flattened laminae provide a unique opportunity to reduce leaf morphology to a two-dimensional shape. Local features (such as serrations and lobes) and general shape attributes (like length-to-width ratio) can be measured, but other methods also exist to measure leaf shape more globally and comprehensively. A popular method to quantify leaf shape is to place (x,y) coordinates, known as landmarks, on homologous features that are related by descent from a common ancestor on every sample (Bookstein, 1997). The set of landmarks from each leaf can be superimposed by translation, rotation, and scaling using a Generalized Procrustes Analysis (Gower, 1975). Once superimposed, the Procrustes-adjusted coordinates of each shape can be used directly for statistical analyses. Landmark analysis excels in its interpretability, because each landmark is an identifiable feature with biological meaning imparted by the shared homology between samples. Because landmarks are homologous features, their use often reveals genetic and developmental patterns in shape variation (Chitwood et al., 2016a).

Not all leaves have obvious homologous features that can be used as landmarks. Further, when comparing leaves with disparate morphologies (e.g., simple vs. compound leaves), there may not be identifiable homologous points. Nearly all leaves have homologous landmarks at the tip and base, but if there are no other identifiable landmarks, an equal number of equidistant points on each sample between the landmarks can be placed (Langlade et al., 2005). The denser and more numerous such pseudo-landmarks are, the closer they come to approximating the contour itself.

Another method, the use of Elliptical Fourier Descriptors (EFDs), measures shape as a continuous closed contour, and can also be used when homologous features are absent. EFD analysis begins with a lossless data compression method called chaincode, in which the up, down, left, right, and diagonal relationship of each successive pixel to the next is recorded as a chain of numbers, 0–7, so that from this chain the closed contour can be faithfully reproduced (Freeman, 1974). The chain code is decomposed by a Fourier analysis into a harmonic series that is used to quantify an approximate reconstruction of the shape (Kuhl and Giardina, 1982).

Both pseudo-landmarks and EFDs measure leaf shapes for which homologous features that can be used as landmarks are lacking (Bensmihen et al., 2008; Chitwood and Otoni, 2017b). Still, when comparing disparate leaf shapes, unless major sources of shape variance in the data (such as the number of lobes or leaflets) are present in every sample, individual pseudo-landmarks or harmonic coefficients will not correspond between samples in a comparable way useful for analysis. For example, an EFD analysis of the abstract *Monstera* leaf shapes from *La Gerbe* (a work from Henri Matisse’s cut out period) demonstrates this problem: the leaves are similar in shape, but the differing numbers of lobes on each leaf creates a circumstance where the harmonic coefficients do not correspond to comparable features, creating a nonsense morphospace, and preventing statistical analyses (Supplementary Figure S1). The differing number of lobes also inherently prevent landmark-based approaches, as the lobes do not correspond with each other.

Recently, a computer vision method coupled with machine learning was used to classify leaves, with diverse vascular patterns and leaf shapes, into plant families and orders (Wilf et al., 2016). This method uses a visual descriptor to train a classifier. Since cleared leaves are used, this method relies on both internal features like branch points in the vasculature as well as features on the leaf margin. These internal features provide a rich set of information which the authors use to classify 7,597 cleared leaves from up to 29 families and 19 orders up to an accuracy of 72.14%. Computer vision circumvents the problems associated with traditional morphometric methods, above, that used defined features for analysis (either landmarks or harmonics from Fourier-based approaches) that prevent a broad comparison across diverse leaf shapes, and the venation patterns of cleared leaves provide abundant information for these methods to classify leaves.

Cleared leaves, though, are time consuming to prepare compared to simply scanning leaves and analyzing their outlines. It is much easier to sample the immense amount of leaf shape diversity using scans and photographs than preparing cleared leaves. Fossil leaves, too, may have shape information associated with a closed contour, and their venation pattern may not be available for analysis. There needs to be a morphometric method that can accommodate the *closed contours* of the diverse leaf shapes found in nature, and although outlines contain less information than the vasculature of cleared leaves, they potentially provide broader sampling of leaf shape diversity across plants.

To develop a morphometric method that (1) comprehensively measures shape features in leaves, both locally and globally, (2) can compare disparate leaves shapes, (3) is robust against noise commonly found in leaf shape data (e.g., internal holes because of overlapping leaflets or small defects introduced during imaging and thresholding), and (4) can be extrapolated to other plant phenotyping needs (e.g., measuring the branching architectures of roots and trees, the spatial distributions of plants in ecosystems, or the texture of different pollen types; Mander et al., 2013, 2017; Li et al., 2017) we used a persistent homology approach. Persistent homology is a topological data analysis method. Topology is the field of mathematics concerned with properties of space (“homology groups”) preserved under deformations (e.g., bending) but not tearing or re-attaching (we stress that “homology” refers to unrelated concepts in biology and topology and that “persistent homology” does not refer in any way to “homology” in the biological sense of the word). Persistent homology measures topological features as a filtration across simplicial complexes (or more simply, a method to measure topological features of spaces across different spatial resolutions; Edelsbrunner and Harer, 2008; Weinberger, 2011; Li et al., 2017).

Here, we present a morphometric technique based on topology, using a persistent homology framework, to measure the outlines of leaves and classify them by plant family. We analyze 182,707 leaves (freely available to download; Chitwood, 2017a), from both published studies and shapes analyzed for the first time, from 141 plant families and 75 sites throughout the world. We first compare the diverse shapes represented in a common morphospace using persistent homology, which captures traditional shape descriptors in varying combinations and novel morphological features, as well. Major phylogenetic groups of plants occupy distinct regions of the morphospace and we estimate plant families that have the most and least diverse leaf shapes. Using persistent homology, we then use a linear discriminant analysis (LDA) to classify leaves by plant family. Persistent homology predicts family at a rate above chance and 2.7 times the rate of traditional shape descriptors. Persistent homology, by measuring topological features, can be generally applied to branching architectures found in plants, providing a shared framework to quantify the plant form comprehensively.

## Results

### Dataset and a Morphospace Defined Using Traditional Shape Descriptors

To broadly analyze seed plant leaf shape diversity collected from sites throughout the world, we used both published and unpublished data. In total, 182,707 leaves were analyzed (**Table 1**). Many of these datasets address specific genetic and developmental questions, focusing on genetic variability within a group or closely related species. Leaves were analyzed from the following groups of plants: *Alstroemeria* (2,392 leaves), apple (9,619 leaves), *Arabidopsis* (5,101 leaves), *Brassica* (1,832 leaves), *Capsicum* (3,277 leaves), *Coleus* (34,607 leaves), cotton (2,885 leaves), grapevine and wild relatives (20,121 leaves), *Hedera* (common ivy, 865 leaves), *Passiflora* (3,301 leaves), Poaceae (866 leaves), wild and cultivated potato (1,840 leaves), tomato and wild relatives (82,034 leaves), and *Viburnum* (2,422 leaves) (please see **Table 1** for references and AUTHOR CONTRIBUTIONS for unpublished data). We also analyzed two datasets that sample broadly across seed plants and from sites throughout the world. The Leafsnap dataset, with 5,733 leaves, represents mostly tree species of the Northeastern United States, but other groups of plants as well (Kumar et al., 2012). The Climate dataset, with 5,812 leaves total, analyzes the relationship between leaf shape and present climates as indicators of paleoclimate (Huff et al., 2003; Royer et al., 2005; Peppe et al., 2011).

**Table 1:** Leaf counts, references, and AUTHOR CONTRIBUTIONS for each dataset.

Leaf type	Count	References and authors
*Alstroemeria*	2392	Chitwood et al., 2012c
Apple	9619	Migicovsky et al., 2018
*Arabidopsis*	5101	AB, RA, CB, ER, BZ
*Brassica*	1832	HA, SG, JP
*Capsicum*	3277	TH, AVD
Climate	5812	Huff et al., 2003; Royer et al., 2005; Peppe et al., 2011
*Coleus*	34607	VC, MF, ML
Cotton	2885	Andres et al., 2017
Grapevine	20121	Chitwood et al., 2014, 2016a,b; VC, MF, LK, JL, AM
*Hedera*	865	Martinez et al., 2016
LeafSnap	5733	Kumar et al., 2012
*Passiflora*	3301	Chitwood and Otoni, 2017a,b
Poaceae	866	LC, TG, PK
Potato	1840	DF, SJ
Tomato	82034	Chitwood et al., 2012a,b, 2013
*Viburnum*	2422	Schmerler et al., 2012; MD, EE, SS, ES
Total	182707	NA


**FIGURE 1:**
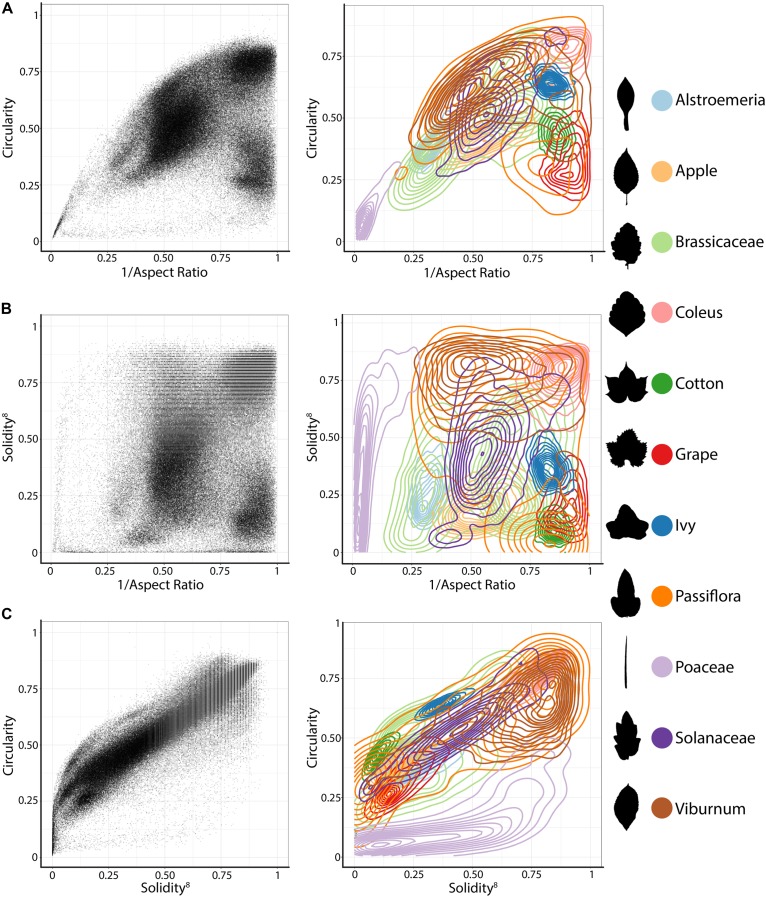
Traditional shape descriptors delimit leaves from different taxonomic groups. **(A)** Circularity vs. 1/Aspect Ratio, **(B)** Solidity^8^ vs. 1/Aspect Ratio, and **(C)** Circularity vs. Solidity^8^. **(Left)** Scatter plots of 182,707 leaves analyzed, from 141 plant families from 75 sites throughout the world. **(Right)** For select taxonomic groups, density plots showing ability of traditional shape descriptors to delimit different leaf shapes and distributions of different groups. Solidity and Aspect Ratio values have been transformed to yield more even distributions. Taxonomic groups are indicated by color and silhouettes of representative leaves close to the overall mean of descriptor values provided.

We analyzed all leaves using the traditional shape descriptors circularity, aspect ratio, and solidity (**Figure 1**). These shape descriptors are simple in the sense that they each measure a very specific aspect of shape, but they are powerful in that they can be applied to any shape, which is not necessarily true of other methods that measure shape more comprehensively (such as landmarks, pseudo-landmarks, and EFDs). Circularity is a ratio of area to perimeter (true perimeter, excluding holes in the middle of the object) measured as $4\pi *\left(\frac{area}{{perimeter}^{2}}\right)$ and is sensitive to undulations (like serrations, lobes, and leaflets) along the leaf perimeter, but is also influenced by elongated shapes (like grass leaves) when comparing leaves with such different shapes, as in this analysis. Aspect ratio is measured as (major axis/minor axis) of a fitted ellipse, and it is a robust metric of overall length-to-width ratio of a leaf. Solidity is measured as $\frac{\text{area}}{\text{convex hull}}$ where the convex hull bounds the leaf shape as a polygon. Leaves with a large discrepancy between area and convex hull (such as compound leaves with leaflets, leaves with deep lobes, or leaves with a distinct petiole) can be distinguished from leaves lacking such features using solidity.

Differences between groups were visualized as scatterplots and density diagrams (**Figure 1**), using transformed values of aspect ratio (1/_(aspect ratio)_) and solidity (solidity^8^) to create more normal distributions that allow the separation between groups to be better visualized. The linear leaves of grasses (Poaceae, in **Figure 1**, lavender) are perhaps the most distinct group of leaf shapes. The Brassicaceae (light green) are bimodal in their distribution, reflecting entire vs. highly lobed and compound leaves, as well as differences in petiole length. *Passiflora* (dark orange), Solanaceae (purple), and *Viburnum* (brown) exhibit broad, continuous distributions, which like the Brassicaceae reflect the diversity of leaf shapes in these groups. *Alstroemeria* (light blue), apple (light orange), *Coleus* (pink), cotton (dark green), grapevine (red), and common ivy (dark blue) all have more localized distributions in the morphospace, indicating that shape variation is expressed within a smaller range, relative to other groups, as measured using traditional shape descriptors.

### Persistent Homology and Non-linear Relationships With Traditional Shape Descriptors

Although traditional shape descriptors can describe important shape features across diverse leaves, they do not measure shape comprehensively like landmarks, pseudo-landmarks, and EFDs. Comprehensive morphometric methods, however, cannot be applied across diverse shapes, only between leaves with similar shapes, as in natural variation studies. We crafted a persistent homology method to quantify the features of leaves. Persistent homology is a Topological Data Analysis method that examines (1) topological features as a (2) filtration across a simplicial complex (Munch, 2017). In the specific case we have devised to measure leaf shape, the topological features are simply “blobs,” contiguous non-touching islands that are “born” and “die” across the filtration. The filtration is a function projected onto the shape. In this case, the filtration is a density function indicating the concentration of pixels. As the filtration passes from high to low density values, “blobs” are “born” and “die” and these features are monitored in the form of a Euler characteristic curve. The details of how persistent homology are implemented here to measure leaf shapes are described in detail, below.

**FIGURE 2:**
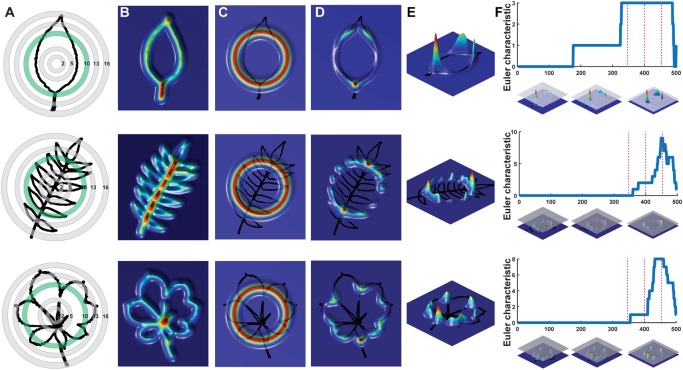
Persistent homology and leaf shape. **(A)** Contours of a simple leaf **(Top)**, compound pinnate leaf **(Middle)**, and compound palmate leaf with a hole and overlap in leaflets **(Bottom)**. 16 annuli used to isolate pixel density are shown, with annulus 10 shown in subsequent panels indicated in green. **(B)** Colormap of a Gaussian density estimator that is robust to noise. Red indicates a larger density of neighboring pixels and blue less density. **(C)** An annulus kernel is used to localize and smoothen data. **(D)** Multiplication of the annulus kernel with the density estimator isolates density features of the leaf contour. **(E)** Side view of the annulus kernel-isolated density features of the leaf. The high peaks in red indicate higher pixel density. **(F)** A plane traverses the density function from the highest to lowest densities (*x* axis). As the plane traverses the function, the topological space is recorded as the number of connected components minus the number of holes above the plane at any given point, the Euler characteristic (*y* axis). Three pink dotted lines correspond to the plane at three points along the density function, which are visualized below the graphs. Together, similar curves from the 16 annuli comprise the persistent homology description of leaf shape.

We begin by conceptualizing shape as a two-dimensional point cloud of an outline defined by pixels (Li et al., unpublished; Migicovsky et al., 2018). A Gaussian density estimator, assigning each pixel a value that indicates the density of neighboring pixels, is calculated (**Figure 2**). In leaves, high density pixels with lots of neighbors tend to reside in the sinuses of serrations or lobes or at points of intersection, such as the attachment points of leaflets to the rachis of a compound leaf. Using a Gaussian density estimator, rather than focusing on continuity of a closed contour (as in pseudo-landmarks and EFDs), minimizes the impact of internal or non-continuous features, such as holes or occlusions made by the overlap of leaflets and lobes (see the bottom palmately shaped leaf in **Figure 2**). Sixteen annuli (or, ring structures) emanating from the centroid of the shape (**Figure 2A**) serve to partition the leaf into subsets of features, increasing the ability to distinguish between shapes. An annulus kernel for each ring (**Figure 2C**) is multiplied by the density estimator (**Figure 2B**) to isolate density features that intersect with the annulus (**Figures 2D,E**). The resulting density function from each annulus is the function across which topological space is measured. As shown in **Figure 2F**, beginning with the highest density level (that is, those pixels with the highest value of the Gaussian density estimator, as shown by the plane intersecting the red in the graphs below the curves in **Figure 2F**, the plane intersecting lower density levels depicted in blue going from Left to Right), the number of connected features with densities above that level is recorded. Counting the number of connected components minus the number of holes (which is a topological feature, known as the Euler characteristic) continues across the function, proceeding to lower density levels. The value of the curve (*y* axis in **Figure 2F**) at each density level (*x* axis in **Figure 2F**) records the topological structure across the values of the function, the crux of persistent homology. A curve is recorded for each annulus, so that using our method, the shape of a single leaf is represented by 16 curves.

**FIGURE 3:**
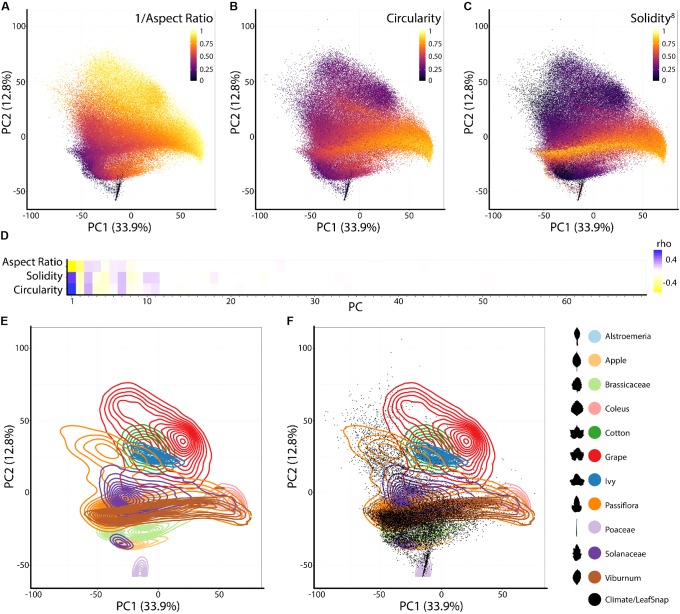
Principal component analysis (PCA) of persistent homology results. Principal Component 2 (PC2) vs. PC1 based on persistent homology results for 182,707 leaves colored by **(A)** 1/Aspect Ratio, **(B)** Circularity, and **(C)** Solidity^8^. Aspect Ratio and Solidity values have been transformed to yield more even distributions. Note non-linear relationships between traditional shape descriptors and persistent homology PCs. **(D)** Correlations between aspect ratio, circularity, and solidity and PCs 1–69 (representing 90% of variation). Positive and negative Spearman’s ρ-values are indicated as blue and yellow, respectively. **(E)** Density plots show distributions of selected taxonomic groups in persistent homology PCA and **(F)** Climate and Leafsnap datasets, representing 141 plant families from 75 sites throughout the world, are superimposed as black dots. Taxonomic groups are indicated by color and silhouettes of representative leaves close to the overall mean of descriptor values provided.

To analyze the persistent homology output, we discretize each Euler characteristic curve into 500 values (**Figure 2F**) and then concatenate these values over the 16 annuli, representing each leaf shape as 8,000 values. A principal component analysis (PCA) performed using the 8,000 values creates a leaf morphospace defined by persistent homology (**Figure 3**). To interpret this morphospace, we colored data using traditional shape descriptor values. Although clear patterns among aspect ratio (**Figure 3A**), circularity (**Figure 3B**), and solidity (**Figure 3C**) with persistent homology data are evident, the relationships are non-linear compared to the orthogonal PC axes. Aspect ratio, circularity, and solidity are similarly correlated with PC1 (ρ-values of -0.72, 0.70, and 0.61, respectively) demonstrating that persistent homology PCs can capture distinct attributes of shape simultaneously (**Figure 3D**). The correlations between traditional shape descriptors and persistent homology PCs rapidly diminish among high order PCs (**Figure 3D**). The non-linear relationship between traditional shape descriptors and persistent homology PCs indicates that persistent homology captures differing combinations of traditional shape descriptors (and novel features) in different ways among the represented leaf shapes. Such non-linear relationships are influenced by the different groups represented in the dataset (**Figure 3E**). If the Leafsnap and Climate datasets—representing 141 plant families and 75 sites from throughout the world—are superimposed as points on top of the independent dataset representing natural variation within a limited number of different groups (**Figure 3F**), then the overall shape of the persistent homology space defined by specific groups is recapitulated, suggesting that the *overall* shape and density of the persistent homology morphospace is partially saturated. This does not mean that there is no other significant leaf shape variation to be explored, only that some archetypal leaf shapes are well represented in our dataset. The boundaries of the persistent homology morphospace allow for speculation. Likely the morphospace is (1) bimodal, defined by elongated leaf shapes found in some Poaceae and Gymnosperms (specifically Pinophyta in the Leafsnap and Climate datasets) compared to other leaf shapes and (2) is defined by variation spanning entire to deeply lobed (or even compound) leaf shapes, as represented by *Passiflora*, Solanaceae, and *Viburnum* across PC1. Of course, other leaf shape variation exists (and is even visually apparent in the plots of PC2 vs. PC1) and other PCs in this dataset remain to be explored. The dataset does not come near to sampling all existing leaf shapes.

### Differences in Leaf Shape Between Phylogenetic Groups and the Most Diverse Plant Families

**FIGURE 4:**
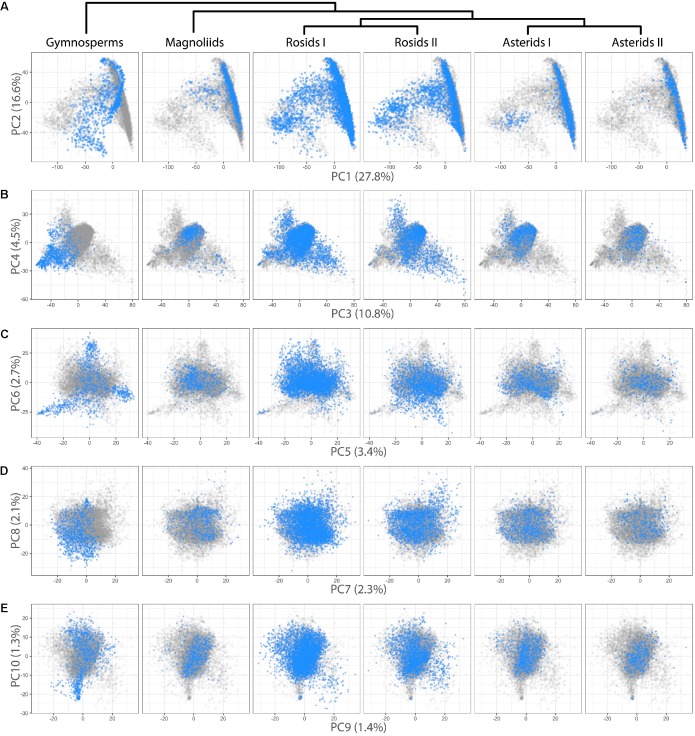
Differences in leaf shape between phylogenetic groups. Gymnosperm, magnoliid, rosid I, rosid II, asterid I, and asterid II leaves **(Left–Right)** are each plotted in blue against all samples (gray) for **(A)** PC2 vs. PC1, **(B)** PC4 vs. PC3, **(C)** PC6 vs. PC5, **(D)** PC8 vs. PC9, and **(E)** PC10 vs. PC9. Percent variance explained by each PC is indicated.

We were interested in detecting difference in leaf shape between phylogenetic groups and performed a PCA for just the Leafsnap and Climate datasets (**Table 1**), which together represent 141 plant families, but without the over-representation from specific taxonomic groups presented earlier. Visualizing gymnosperms, magnoliids, rosids I, rosids II, asterids I, and asterids II across PCs 1–10 (representing 73% of shape variance) clear differences in persistent homology shape space can be detected (**Figure 4**). Differences in shape are most easily detected for the earliest diverging lineages. For example, gymnosperms occupy a distinct region of morphospace defined by PCs 1–6 (**Figures 4A–C**) compared to angiosperms. Subtler differences between recently diverging groups can also be seen. Asterids II, for example, are excluded from some regions of morphospace occupied by rosids I/II and asterids I for PCs 1–4 (**Figures 4A,B**).

**FIGURE 5:**
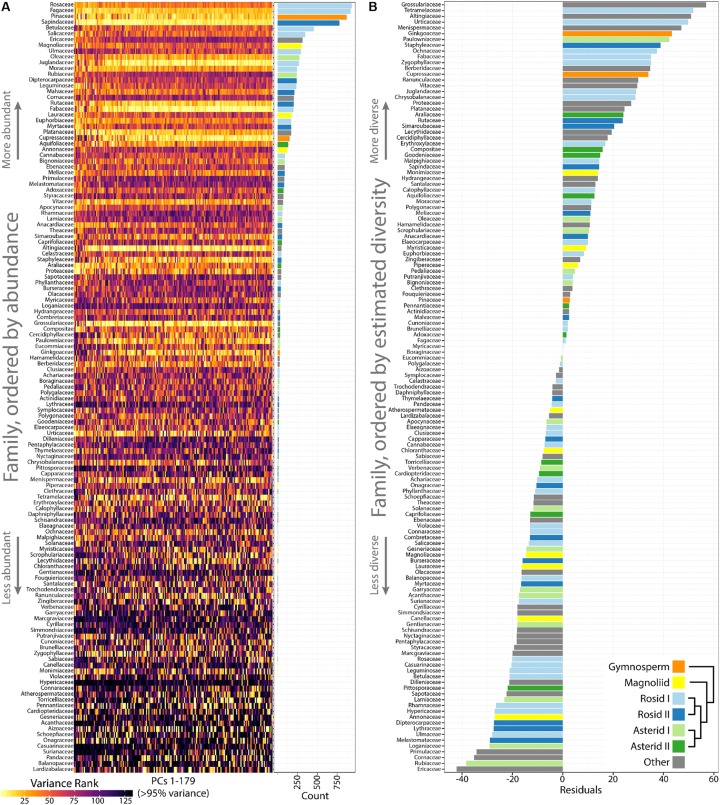
Highly variable plant families are variable across Principal Components (PCs) and estimates of leaf shape diversity by family. **(A)** Variance was measured for each plant family and then ranked from most variable (yellow) to least variable (black) for each PC. Plant families are ordered by abundance, as seen in the bar graph **(Right)** indicating count number in the dataset. The most abundant plant families in the dataset tend to be the most variable. **(B)** Linear regression was used to model the -median variance ranking for each plant family as a function of log_10_(count). The residuals are estimates of plant family leaf shape diversity, as corrected for representation in the dataset. Higher residual values indicate higher estimated leaf shape diversity. Gymnosperms, orange; magnoliids, yellow; rosids I, light blue; rosids II, dark blue; asterids I, light green; asterids II, dark green; other groups, gray.

Differences in occupied morphospace between phylogenetic groups prompted us to ask: are plant families diverse across all PCs or just some, and what are the most and least morphologically diverse families? To answer the first question, we calculated variance across PCs 1–179 (representing >95% of all shape variance) for each plant family and then ranked families from most to least variable for each PC (**Figure 5A**). Visualizing the ranked variability of families across PCs (where PCs are color coded from the most variable, yellow, to the least, black; **Figure 5A**), it is apparent that the most diverse families tend be the most diverse across all PCs. Increased variability in persistent homology PCs, though, might simply be due to more leaves in some families compared to others. Indeed, the most diverse plant families are also the most represented in our dataset, as seen when families are arranged by abundance (**Figure 5A**, see bar graph of counts on the Right side). Because highly variable families tend to be variable across PCs, we took the median rank of variance across PCs as a measure of overall family leaf shape diversity. The relationship between –median rank variance and log_10_(count) is linear (Supplementary Figure S2). Using linear regression, we took the residuals from the model as an estimate of plant family leaf shape diversity, corrected for differences in sample size (**Figure 5B**). A wilcoxon signed rank test on residuals indicates that asterids I are marginally significant (*p* = 0.08) for lacking diversity (two sided, μ = 0) but other groups (gymnosperms, *p* = 0.25; magnoliids, *p* = 0.20; rosids I, *p* = 0.97; rosids II, *p* = 0.63; asterids II, *p* = 0.63) show no detectable biases in diversity. The overall results indicate that, for the current dataset, leaf shape diversity within major phylogenetic plant groups is equivalent, but specific families have higher estimated leaf shape diversity than others.

### Persistent Homology Predicts Plant Family and Outperforms Traditional Shape Descriptors

**FIGURE 6:**
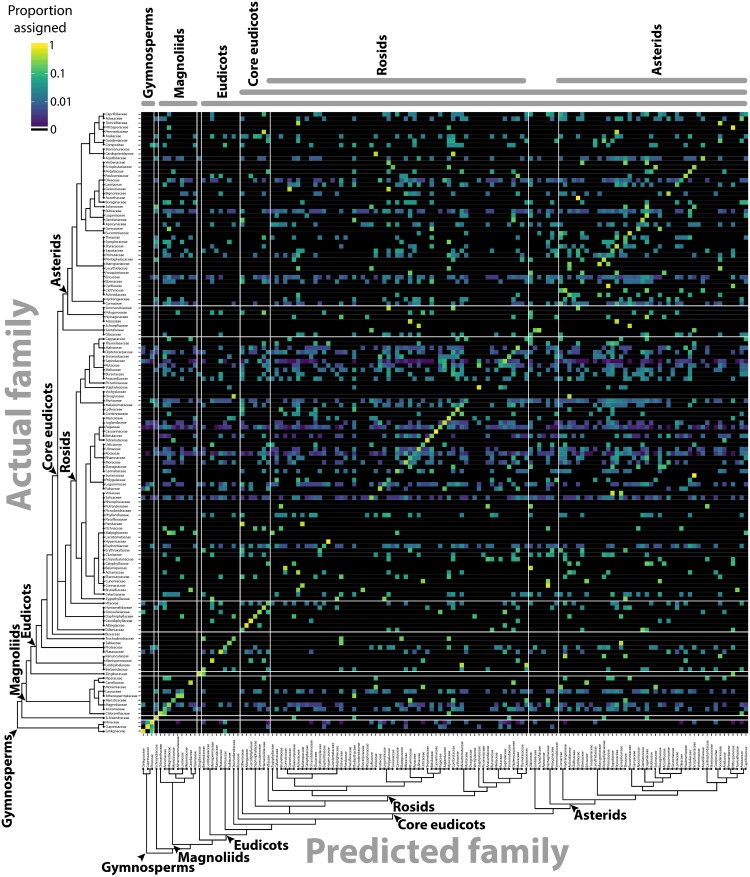
Predicting plant family using persistent homology. Using persistent homology data from the Climate and Leafsnap datasets, a linear discriminant analysis (LDA) was used as a classifier to predict plant family, cross-validated using a jackknifed “leave one out” approach. The vertical axis indicates actual plant family and the horizontal axis predicted plant family. Color indicates proportion of leaves from each actual plant family assigned to each predicted family, such that proportions across the horizontal axis sum to 1. Black indicates no assignment. A phylogeny indicating key taxonomic groups is provided.

The separation of different groups in the traditional shape descriptor (**Figure 1**) and persistent homology (**Figures 3**, **4**) morphospaces suggests the ability to predict the phylogenetic identity of a leaf based on its shape. Previous computer vision approaches coupled with machine learning have successfully predicted plant family and order using vein patterning and margin features (Wilf et al., 2016). Can the same be done using a persistent homology analysis of the outline alone? Using the Leafsnap and Climate datasets (**Table 1**) that together represent 141 plant families, we used a LDA on PCs 1–179, representing >95% of the persistent homology morphospace variation, to create a classifier scheme. Leaves were then reassigned to the linear discriminant space using a cross-validated “leave one out” approach (Venables and Ripley, 2002) and the results visualized as a confusion matrix (**Figure 6**), plotting the actual family identity of leaves as a function of the proportion of their predicted family identity. Family was used as the taxonomic level of prediction because it was the most specific level of identification common to all leaves. Using persistent homology, there was a 27.3% correct plant family assignment rate of leaves. Using a bootstrapping approach permuting plant family identity against leaf shape information, a 27.3% correct reclassification rate or higher was never achieved in 1,000 bootstrapped simulations, indicating that assignment is above chance. This outperforms traditional shape descriptor prediction (at a rate of 10.2%) by 2.7 times (**Table 2**), and including both persistent homology and traditional shape descriptor data only marginally increases the prediction rate (to 29.1%) over that of persistent homology alone (27.3%), indicating that persistent homology largely captures the same shape features as traditional descriptors, but provides additional information as well. If order is used as the taxonomic level of prediction, prediction rates are almost identical to those for family (27.3% for persistent homology, 9.2% for traditional shape descriptors, and 29.1% for both).

**Table 2:** Overall prediction rates of plant family using different morphometric methods.

Method	Correct
Persistent homology	27.3%
Traditional descriptors	10.2%
Both methods	29.1%


## Discussion

We have presented a new morphometric method using persistent homology, a topological approach, that can comprehensively measure leaf shape. Other methods measure leaf shape comprehensively, including traditional landmarks, pseudo-landmarks, and EFDs. However, no method comparatively analyzes the diverse *shapes* of leaves in seed plants (simple leaves, deeply lobed leaves, compound leaves of different shapes, leaves with differing numbers of leaflets or lobes, or large variation in petiole length and shape), only naturally varying leaves among related plant species (Supplementary Figure S1). Other morphometric methods that only analyze the external contour of shapes are sensitive to artifacts, such as internal holes made by the overlap of leaflets or lobes, or small errors during thresholding and isolation. Finally, although appropriate for plant organs that can be represented by discrete shapes—like leaves, petals, seeds, or other lateral organs—current morphometric techniques fail to capture other attributes of plant architecture, like the branching patterns of roots, shoots, and inflorescences. A framework that can not only measure shape, but other features that are important to the plant form, is currently lacking.

**FIGURE 7:**
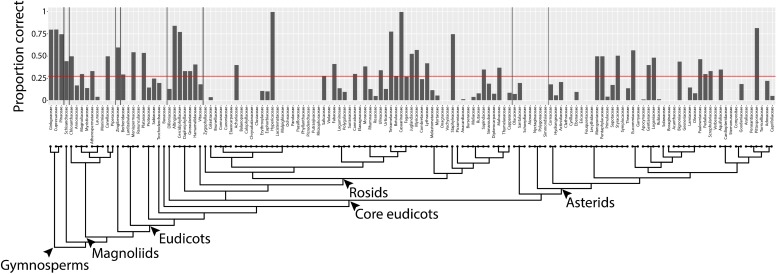
Prediction rates using persistent homology data across plant families. Proportion of leaves from each family correctly assigned. Red line indicates overall correct prediction rate of plant family of 27.3%. Phylogeny and major taxonomic groups are indicated.

By converting shapes into a topological space, as defined by a function that isolates subsets of the shape and describes it in terms of neighboring pixel density (**Figure 2**), the described persistent homology approach can compare disparate leaf shapes across seed plants, allowing for the approximation of the overall leaf morphospace (**Figure 3**). By estimating pixel density, the method accommodates internal features (such as holes caused by leaflet overlap) or small processing artifacts, that do not unduly influence the output compared to the absence of such imperfections. The ability to compare shapes broadly and be robust against processing artifacts will enable large scale data analyses in the future, such as the analysis of digitized herbarium vouchers, ecological studies, or genetic and developmental insights into complex morphologies, for which current morphometric approaches are not designed. We detected clear differences in leaf shape between major phylogenetic groups (**Figure 4**) and estimated leaf shape diversity across plant families (**Figure 5**), demonstrating that a persistent homology approach is relevant for large-scale morphometric studies across plant evolution. The ability to comprehensively measure shapes permits alternative statistical approaches, moving beyond descriptive statistics used with traditional shape descriptors (**Figure 1**) and allowing for classifier and prediction approaches (**Figures 6**, **7** and **Table 2**). Although the overall prediction rate of 27.3% for plant family is relatively low (**Table 2**), it is important to remember that it is above the level of chance (determined by bootstrapping, 1,000 simulations) and that the rates are not evenly distributed across family. Plant family prediction rates vary from 0 to 100% (**Figure 7**). The variability in rates is not overly influenced by sampling depth or variation within a group. For example, prediction rate of plant family and abundance are correlated at ρ = 0.37, and the correlation with median rank PC variance is ρ = -0.24. It is also important to keep in mind that plants are usually identified using floral structures, and leaf morphology is not the most discriminating morphological factor between species. Additionally, our prediction is distributed over 141 plant families, whereas previous studies were predicting over fewer, which decreases the correct assignment rate. Although comprehensive, our dataset does not begin to encompass the total shape variation present in a plant family and there are undoubtedly collection biases in the data influencing prediction. Other factors than diversity within a group or the degree to which it is sampled, though, likely influence prediction rate too.

Theoretically, a unifying morphometric framework that can accommodate not only shapes but the branching architectures of plants, is lacking. Topology is a field of study concerned with features that are invariant under deformations, such as bending or stretching. Traditional morphometric methods (like landmarks and EFDs) work superbly with shapes that have either homologous features for landmarks or features that allow pseudo-landmarks and harmonic coefficients to correspond (some types of leaves, seeds, petals, etc.). Computer vision, machine learning, and deep learning approaches work especially well with two-dimensional gray-scale and color image data for prediction and identification (Joly et al., 2016; Wilf et al., 2016).

But the plant form is not a shape or a two-dimensional color image. Plants are branching architectures that develop through time. The connectedness of branches—irrespective of deformation or bending—is topology, and it is useful for describing variation in plant architecture (Li et al., 2017). Describing branching patterns is relevant to describing phylogenetic trees, too, to which Topological Data Analysis approaches have been applied (Munch and Stefanou, 2018). We converted shape into a topological feature space to comprehensively describe leaf shape diversity where other methods have failed. But separate from this use (for shapes) persistent homology is a promising technique to describe diverse plant structures, including root architecture (Li et al., unpublished), serrations and branching patterns, and venation architecture in novel ways, quantifying complex morphological features relevant to botany and taxonomy that previously have only been described qualitatively. Topological Data Analysis and persistent homology approaches can also be applied in *n-*dimensional space. Plant development occurs in 4D: 3D, over time. Rather than describing development as a time series, plant morphology can be quantified as it truly is—a single, four dimensional shape. There is a place for measuring plant morphology in terms of topological features, and this space has not been thoroughly explored yet, and can potentially drive a more comprehensive analysis of plant architecture across diverse cells, tissues, organs (pollen textures; Mander et al., 2013), organismal forms, or biomes (satellite images of the savannah; Mander et al., 2017), as we have used it here for leaves. The morphometric approach described here is compatible with similar persistent homology methods, creating a shared framework in which the plant form can be measured (Li et al., unpublished).

## Materials and Methods

### Leaf Shapes

The 182,707 leaf outlines from 141 plant families from 75 sites throughout the world used in this manuscript are available to download (Chitwood, 2017a). This file directory includes *x*,*y* coordinates that form the outlines of the leaves. Separate folders contain text files with *x*,*y* coordinates for the leaves from each of the indicated groups in **Table 1**. Within each folder, original *x*,*y* coordinates and scaled coordinates are provided. This dataset contains leaves from both published and unpublished sources (see **Table 1** for details).

### Persistent Homology

The MATLAB code necessary to recapitulate the persistent homology analysis in this manuscript can be found in the following GitHub repository (Li, 2017)^1^.

Persistent homology is a flexible method to quantify branching structures (Edelsbrunner and Harer, 2008; Weinberger, 2011; Li et al., 2017), point clouds (Ghrist, 2008), two-dimensional and three-dimensional shapes (Gamble and Heo, 2010), and textures (Mander et al., 2013, 2017). Each of these different phenotypes can be described by a multidimensional vector (e.g., Euler characteristic curve), integrating how homology (e.g., path-connected components) persists across the filtration of a simplicial complex.

Leaf contours are represented as two-dimensional point clouds extracted from binary images (**Figure 2A**). We use a Gaussian density estimator, which can be directly derived from the point cloud and is also robust to noise, to estimate the neighborhood density of each pixel. Denser point regions, such as serrations, lobes, or the attachment points of leaflets, have higher function values (**Figure 2B**). Formally, the Gaussian density estimator is defined as

$$\Phi (x):=\frac{1}{n}\overset{n}{\underset{i=1}{\Sigma }}\frac{1}{\sqrt{2\pi }}e^{-\frac{1}{2}{\left(\frac{x-y_{i}}{h}\right)}^{2}},$$

where y_1_, … ,y_n_ are the data points and h is a bandwidth parameter. Because a set of local and regional topologies may often be more effective to represent shapes, we use a local persistent homology technique to subset the density estimator into 16 concentric annuli centered around the centroid of the leaf (**Figures 2A,D**). To achieve this, we multiply this function by a “bump” function *K* which highlights an annulus, defined as

$$K_{\sigma ,t,y}(x):=e^{-\frac{{\left(d(x,y)-t\sigma \right)}^{2}}{{2\sigma }^{2}}},$$

where *y* is the center of the annulus, *t*σ determines its radius, and the parameter σ is its width (**Figure 2C**). Each local function emphasizes the density function falling in the annulus. Given a threshold and a local function, the points whose function values are greater than this threshold form a subset (superlevel set). Changing this threshold value from the maximum function value to its minimum value, we can get an expanding sequence of subsets, or a superlevel set filtration. **Figure 2E** shows the shapes above a plane, an example of a superlevel set filtration. For each subset, we calculate the Euler characteristic, which equals the number of connected components minus the number of holes. Thus, for a sequence of subsets, we get a sequence of numbers (a multidimensional vector). All 16 annuli derive 16 multidimensional vectors which are concatenated into an overall vector used for analysis. PCA was performed in MATLAB on the vectors and PC scores and percent variance explained by each PC used in subsequent analyses.

### Statistical Analysis and Visualization

The R code (R Core Team, 2017) and data necessary to recapitulate the statistical analyses and figures in this manuscript can be found as a zipped folder directory on figshare (Chitwood, 2017b)^2^.

Unless otherwise specified, all graphs were visualized using ggplot2 (Wickham, 2016). Scatterplots were visualized using the geom_point() function, density plots were visualized with the geom_density2d() function, heatmaps were visualized using the geom_tile() function, and colors were selected from ColorBrewer (Harrower and Brewer, 2003) and viridis (Garnier, 2017). Other visualization functions used and specific parameters that can be found in the code used to generate the figures (Chitwood, 2017b).

Variance was calculated for each plant family for each principal component using var() and families ranked for each principal component using rank() (**Figure 5**). Linear regression was performed using lm() and residuals retrieved to estimate leaf shape diversity for each plant family (Supplementary Figure S2). The Wilcoxon signed rank test was performed using wilcox.test() to test for higher or lower than expected phylogenetic diversity using a two-sided test with μ = 0. LDA was performed using the lda() function in the package MASS (Venables and Ripley, 2002). LDA was performed using the number of principal components that contributed at least 95% of all variance (PCs 1–179 for phylogenetic prediction). Prediction using the discriminant space was performed using CV = TRUE for a “leave one out” cross-validated jack-knifed approach and the priors set equal across factor levels. Prediction rates were bootstrapped over 1,000 simulations. A for loop was used, permuting family against leaf identity, performing an LDA on the permuted data, and recording the correct prediction rate for each permuted simulation. A permuted correct prediction rate (out of 1,000 simulations) higher than the actual correct prediction rate was never detected.

## Author Contributions

HA, RA, CB, AB, LC, VC, MD, EE, DF, HF, MF, TG, SG, TH, SJ, BK, PK, LK, VK, JL, ZM, AM, RM, SM, WO, JP, ER, SS, ES, CT, AVD, KZ, LZ, and BZ contributed leaf shape data and read the manuscript. ML provided the analysis. ML and DC wrote the manuscript.

## Conflict of Interest Statement

The authors declare that the research was conducted in the absence of any commercial or financial relationships that could be construed as a potential conflict of interest.

## Footnotes

1 https://github.com/maoli0923/Persistent-Homology-All-Leaf

2 https://figshare.com/articles/LeafMorphospace/4985561/1
