# Supplementary figures and images for: Topological Data Analysis as a Morphometric Method: Using Persistent Homology to Demarcate a Leaf Morphospace
Source: Front Plant Sci. 2018 Apr 25;9:553. doi: 10.3389/fpls.2018.00553 (PMC5996898; doi:10.3389/fpls.2018.00553)

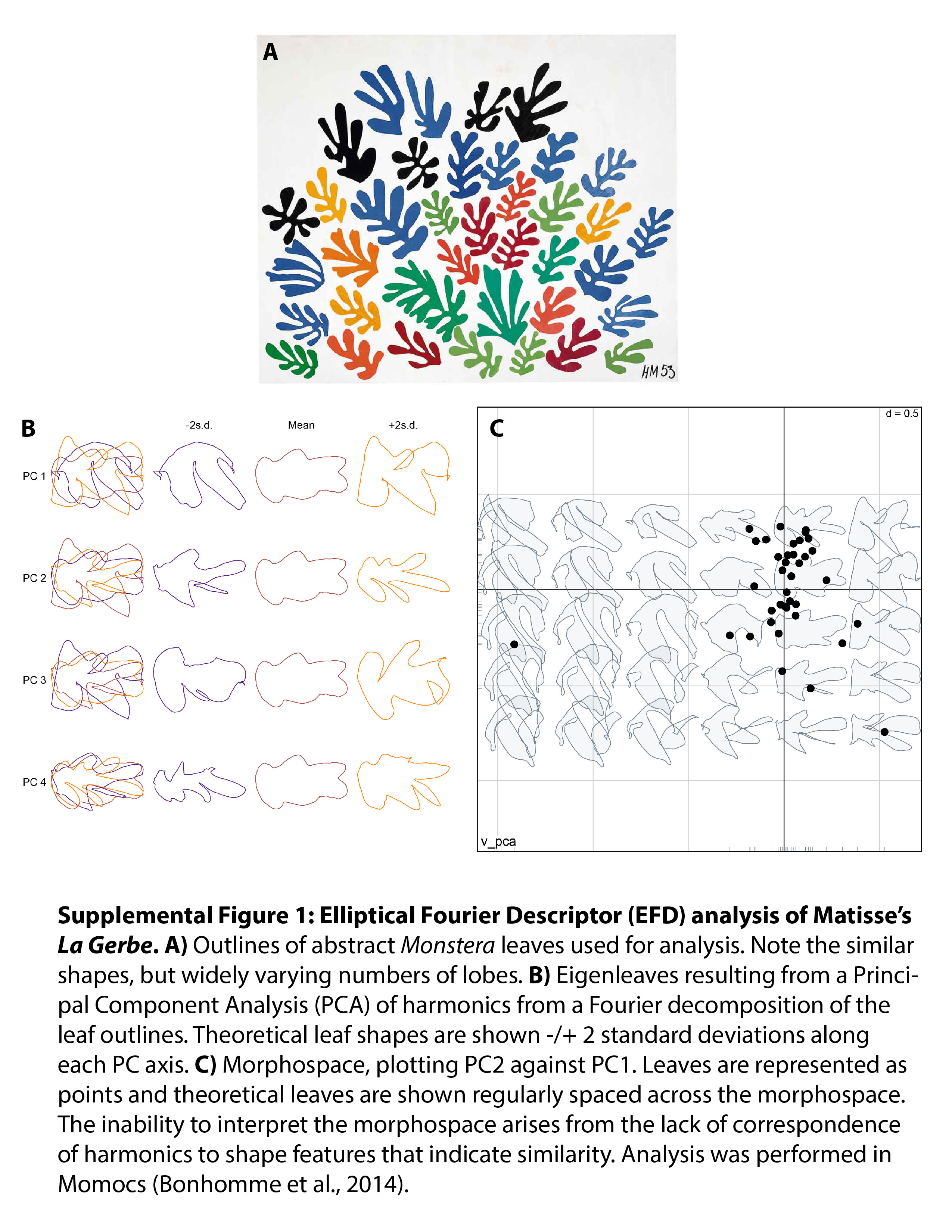

Supplement: Supplementary file 1 [file Image_1.JPEG]

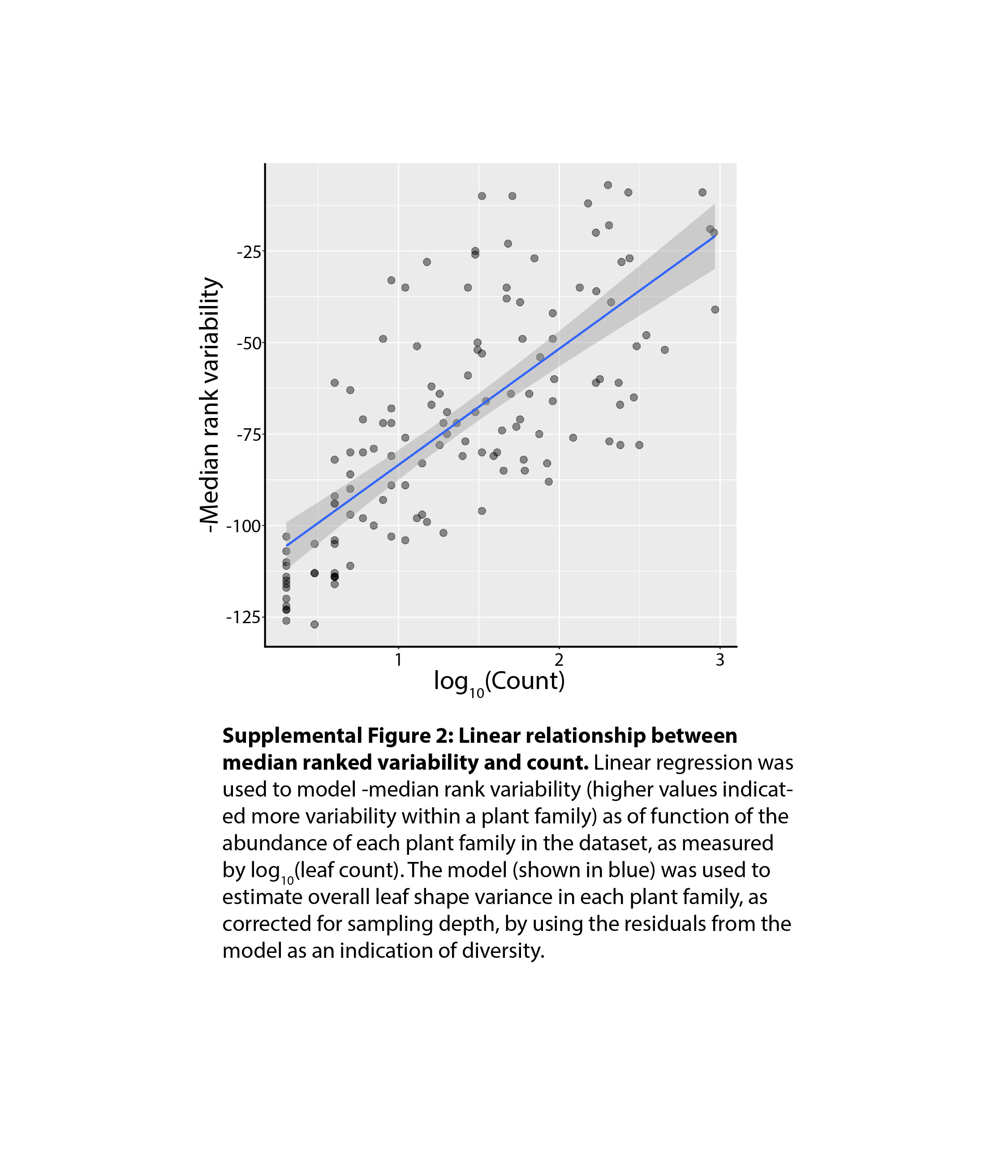

Supplement: Supplementary file 2 [file Image_2.JPEG]
